# Cases of Aneurism Cured by Compression

**Published:** 1849-11

**Authors:** 


*Cases of Aneurism Cured by Compression.*—Dr. Edward Hutton has communicated to the *Dublin Medical Press* (May 16th), the two following cases of aneurism successfully treated by compression:—

Case 1.—James Collins, aet. 34, a farm servant, was admitted into the Richmond Hospital on the 22d of July, 1848, for aneurism of the left brachial artery. He stated that two months previously he was bled in the left arm by a "country bleeder" for a pain in his chest. He remarked at the time that the blood spurted out to a great distance, and was of a bright red color. There was much difficulty in stopping the bleeding, and the bandage was put on very tightly. In three days he removed it, and found that the external wound had healed, but beneath this he observed a small pulsating tumor. He, however, returned to his labor, and continued to work for about a month, when he was obliged to desist on account of pain and a sense of weakness in the arm. The tumor had begun to increase a week before this, and pulsated strongly. No treatment was applied until his admission into the hospital; the tumor was then about the size of a pullet's egg, and presented the usual characters of aneurism. When pressure was made on the brachial artery above the tumor, it lost its pulsation and tension, and yielded in some degree to pressure, but did not wholly collapse, indicating the presence of some coagulum in the sac. The skin covering this was of natural color and thickness. The radial and ulnar arteries pulsated distinctly at the wrist. Immediately on his admission into the hospital, the treatment by compression was commenced. The instrument first used was the screw-clamp, and although the pressure was applied occasionally at different points in the course of the artery, and so regulated as only just to stop pulsation, yet he was never but once able to sustain it for four hours together. After using the screw clamp in this manner at intervals for twelve days, with but little effect upon the tumor, it was laid aside, and Dr. Carte's instrument was applied; with this the patient maintained the compression during six hours in succession, and made much less complaint of its application, At the end of this period, all pulsation had ceased, and never returned. The aneurismal tumor did not, however, begin either to diminish in size or to increase in firmness until nearly a fortnight afterwards, when these changes commenced. He was detained in the hospital until the 19th of September. The tumor was then very firm, and about one-fourth smaller. Immediately on leaving the hospital he returned to his labor, and was actively employed in gathering in the harvest. In March, 1849, I heard from Dr. Harkan, of Elphin, and subsequently from the patient himself, that the tumor was very small and firm, that he felt no inconvenience whatever from it, and had the full use of his arm.

Case 2.—Philip Dignam, aet. 22, applied for advice on the 3d of January, 1849, for aneurism of the left popliteal artery. The tumor was about the size of a hen's egg, situated in the lower part of the popliteal space. It pulsated strongly, and presented all the usual signs of aneurism. When the femoral artery was compressed, the tumor became flaccid, and could be emptied of a considerable portion of its contents, but some solid coagulum remained. He stated that, about six months previously, he first felt a "stinging pain" in the ham; this was occasional only, and did not prevent him from following his usual employment. Two months before his application for advice, he first perceived a tumor which was pulsating and painful, and attended with numbness and weakness of the leg. The tumor slowly enlarged to the size mentioned; his general health was good, and he had not confined himself to his house until a day or two before he came under treatment. On the 3d of January, I applied Dr. Carte's compressing apparatus. The patient was informed of the nature of his disease; of the alternative that awaited him if the plan of compression failed; the mode of managing this was explained to him, and he was exhorted to maintain the compression for six or seven hours, or longer if he could. He was very anxious to avoid an operation, and readily undertook the treatment. The next day, January 4th, the pulsation in the tumor had ceased. He reported that he kept up the compression seven hours and a half in succession, and that, during the whole time of its application, no pulsation returned to the tumor, nor did it after the removal of the instrument. The temperature of the leg and foot did not appear to differ sensibly from that of the sound limb, but the thermometer was not applied. It was difficult to feel pulsation distinctly in the tibial arteries of the right leg, and impossible to do so in the left. Perfect rest and moderate diet were enjoined. After a week, some obscure pulsation was perceived, not dilating the tumor, but as if the popliteal artery was previous along its base. Dr. Carte's instrument was, therefore, again applied for three hours, after which this pulsation was no longer to be felt. Two arteries were traced along the surface of the tumor; one about the size of the temporal, the other smaller. The case now progressed favorably, the tumor became very firm, and diminished in size. In less than four weeks from the commencement of the treatment, he returned to his employment in a butter crane, where he was engaged in lifting heavy weights. I have since seen him occasionally. The tumor, when last examined, was about the size of a nut, and of firm consistence. The pulse in the femoral artery could be felt along its course to within two inches of the tumor. He was free from all uneasiness in the leg, and in fact was completely cured.

*Remarks.*—Dr. Carte's application of an elastic force in the compresion of arteries promises in a great measure materially to lessen the pain attending it, and thus to remove the only plausible objection to this mode of treating aneurisms becoming a rule of surgical practice. In the first case related, the patient was very sensitive to pain, and had not fortitude to endure the screw-clamp for the requisite period; while he was able to sustain the elastic force for six hours without shifting the instruments from the artery, and this period proved sufficient for his altimate cure. In the second case, the compression was maintained during seven hours and a half without relaxation, which I am persuaded could not have been borne with the screw-clamp.—*Ohio Med. and Surg. Journal.*

